# Health-related quality of life and long-term prognosis in chronic hypercapnic respiratory failure: a prospective survival analysis

**DOI:** 10.1186/1465-9921-8-92

**Published:** 2007-12-17

**Authors:** Stephan Budweiser, Andre P Hitzl, Rudolf A Jörres, Kathrin Schmidbauer, Frank Heinemann, Michael Pfeifer

**Affiliations:** 1 Center for Pneumology, Donaustauf Hospital, Donaustauf, Germany; 2 Institute and Outpatient Clinic for Occupational and Environmental Medicine, Ludwig-Maximilians-University, Munich, Germany; 3 Department of Internal Medicine II, Division of Respirology, University of Regensburg, Regensburg, Germany

## Abstract

**Background:**

Health-related quality of life (HRQL) is considered as an important outcome parameter in patients with chronic diseases. This study aimed to assess the role of disease-specific HRQL for long-term survival in patients of different diagnoses with chronic hypercapnic respiratory failure (CHRF).

**Methods:**

In a cohort of 231 stable patients (chronic obstructive pulmonary disease (COPD), n = 98; non-COPD (obesity-hypoventilation syndrome, restrictive disorders, neuromuscular disorders), n = 133) with CHRF and current home mechanical ventilation (HMV), HRQL was assessed by the disease-specific Severe Respiratory Insufficiency (SRI) questionnaire and its prognostic value was prospectively evaluated during a follow-up of 2–4 years, using univariate and multivariate regression analysis.

**Results:**

HRQL was more impaired in COPD (mean ± SD SRI-summary score (SRI-SS) 52.5 ± 15.6) than non-COPD patients (67.6 ± 16.4; p < 0.001). Overall mortality during 28.9 ± 8.8 months of follow-up was 19.1% (31.6% in COPD, 9.8% in non-COPD). To identify the overall role of SRI, we first evaluated the total study population. SRI-SS and its subdomains (except attendance symptoms and sleep), as well as body mass index (BMI), leukocyte number and spirometric indices were associated with long-term survival (p < 0.01 each). Of these, SRI-SS, leukocytes and forced expiratory volume in 1 s (FEV_1_) turned out to be independent predictors (p < 0.05 each). More specifically, in non-COPD patients SRI-SS and most of its subdomains, as well as leukocyte number, were related to survival (p < 0.05), whereas in patients with COPD only BMI and lung function but not SRI were predictive.

**Conclusion:**

In patients with CHRF and HMV, the disease-specific SRI was an overall predictor of long-term survival in addition to established risk factors. However, the SRI predominantly beared information regarding long-term survival in non-COPD patients, while in COPD patients objective measures of the disease state were superior. This on one hand highlights the significance of HRQL in the long-term course of patients with CHRF, on the other hand it suggests that the predictive value of HRQL depends on the underlying disease.

## Background

Home mechanical ventilation (HMV) is an established approach in the treatment of severe, chronic hypercapnic respiratory failure (CHRF). The number of patients treated with HMV has much increased and will rise further with medical advance and the ageing of the population [1]. However, the knowledge on clinical outcome measures during current HMV that could be valuable for long-term follow-up and for estimation of survival, is limited [2,3].

In chronic obstructive pulmonary disease (COPD), the degree of airway obstruction insufficiently represents the systemic aspects of the disease [4]. Accordingly, body mass index (BMI) and six-minute walk distance (6-MWD) have been revealed as prognostic markers [5]. Moreover, long-term survival is linked to the patients' perception of functional limitations, expressed as degree of dyspnoea [6]. There is also evidence for an association with health-related quality of life (HRQL) in terms of disease-specific [7,8] or generic measures [7,9]. Intuitively, self-reported health-status has the potential to integrate diverse aspects of disease severity and prognosis [8].

Patients with CHRF might not only suffer from COPD but also from severe restrictive diseases (RD), neuromuscular disorders (NMD), or obesity-hypoventilation syndrome (OHS). In many of these patients nutritional depletion or systemic inflammation is present [10-14] and associated with survival, as in COPD [2,3], while the relationship between other measures and long-term prognosis might be different [15-17]. In addition, psycho-social factors are relevant in these chronic respiratory diseases [18-21] and could also determine long-term survival.

To account for the specific conditions of the disorders underlying CHRF, the Severe Respiratory Insufficiency (SRI) questionnaire has been introduced [22], providing a comprehensive, multidimensional picture. It can be hypothesized that this reflects features that are common in CHRF and related to prognosis, particularly under the relatively stable conditions achieved by HMV. We thus evaluated the association between disease-specific HRQL and long-term survival, comparing its predictive value with that of known risk factors. The analysis was performed in two-step manner, first identifying the role of HRQL in the total study population and then elucidating the role in patients with or without COPD.

## Methods

### Population

Between December 1^st^, 2002, and November 30^th^, 2004, consecutive patients with current nocturnal HMV (since ≥ 3 months) due to CHRF were prospectively recruited during a routine follow-up investigation. The underlying diseases comprised COPD, severe RD, OHS/overlap syndrome or NMD.

All patients were categorized according to their primary diagnosis upon initiation of HMV. The diagnosis of COPD relied on symptoms and airflow limitation (ratio of forced expiratory volume in one second to inspiratory vital capacity (FEV_1_/FVC) < 0.7) [23]. OHS was characterized by BMI > 30 kg/m^2^, daytime arterial carbon dioxide tension (PaCO_2_) ≥ 45 mmHg prior to HMV and symptoms of CHRF in the absence of other significant causes of hypoventilation based on the physician's judgement [15]. Patients with hypercapnia as a result of confirmed sleep apnoea and minor airway obstruction were classified as "overlap syndrome" (OL) [24]. Participants had to be in a stable clinical condition without signs of current exacerbation or respiratory tract infection. The study was approved by the Institutional Review Board of the University of Regensburg and patients gave their informed consent.

### Assessments and protocol

Upon inclusion the SRI questionnaire, blood gases, laboratory parameters and the presence of comorbidities were assessed, as well as lung function measurements performed.

The SRI questionnaire comprises 49 questions across 7 domains covering respiratory complaints (RC), physical functioning (PF), attendant symptoms and sleep (AS), social relationship (SR), anxiety (AX), psychological wellbeing (PW), and social functioning (SF). These subscales are aggregated into one summary score (SRI-SS), whereas high values indicate high HRQL and converse [22]. For data evaluation the values obtained from the questionnaire were scaled from 0 to 100 analogous to the computation of percentages.

Capillary blood gases (Rapidlab; Bayer Inc; East Walpole, MA, USA) were analyzed during spontaneous breathing of room air if possible or otherwise during the patients' usual oxygen flow. Spirometry (MasterScreen, Viasys Inc., Würzburg, Germany) including assessment of (IVC), was performed according to ATS guidelines [25], and ERS reference values [26] were used. Among the available routine laboratory parameters which were obtained by standard procedures, we selected haemoglobin level and leukocyte number for analysis (Micros 60-CT, ABX Inc., Montpellier, France). Additionally, comorbidities as taken from the medical records or diagnosed *de novo *during the initial hospital stay were documented.

### Follow-up

Patients were routinely admitted every 6 months for re-evaluation of their respiratory status. This included the assessment of adverse effects of HMV treatment (leakage, dry mucosal, etc.) and treatment efficacy by a standardized procedure. Pulmonary function test were performed as described above. At this visit also ventilatory parameters were optimized, guided by nocturnal capillary blood gas values and oxygen saturation. Adherence to HMV was evaluated from the time counter readings of the ventilator and the duration of HMV was calculated in months from the day of initiation.

All patients were followed until death or the end of the study period at November 30^th^, 2006. In patients who could not be re-assessed in the hospital until this closing date, vital status was assessed through telephone interview of the patients' relatives and/or family doctors, or by reviewing the medical records supplied by other medical institutions. Deaths from either respiratory or any cause were recorded.

### Statistical analysis

Data for continuous variables are presented as mean ± standard deviation (SD) or as median values and quartiles, depending on whether the data showed normal distribution or not. Groups were compared by analysis of variance (ANOVA) with *post hoc *comparisons according to Newman-Keuls, alternatively the unpaired t-test or the Mann-Whitney U-test for quantitative variables (with appropriate Bonferroni correction), or by Fisher's exact test for binary variables. Univariate survival analysis was performed by Kaplan-Meier analysis (log-rank test), starting by the day of inclusion to the closing date. As cut-off we used median or quartile values. Multivariate Cox regression analysis was employed to identify independent predictors. P-values < 0.05 were considered statistically significant. All analyses were performed by the statistical software packages SPSS (version 12.0, Chicago, IL, USA) and MedCalc (version 9.2.0.1., Mariakerke, Belgium).

## Results

### Patients' characteristics

Of 262 eligible patients to whom the questionnaire was handled, 12 provided incomplete and 10 non-usable answers, while 9 patients rejected the questionnaire. Thus the study population (Table 1) comprised 231 patients (145 male, 86 female) with CHRF due to either very severe COPD (n = 98) of GOLD (Global Initiative for Chronic Obstructive Lung Disease [23]) stage IV, OHS/OL (n = 54/15), RD (n = 49; comprising chest-wall disease (n = 37), post-tuberculosis syndrome (n = 8), lung fibrosis (n = 3), silicosis (n = 1)), or NMD (n = 15).

**Table 1:** Baseline characteristics of patients according to the aetiology of CHRF.

Parameter	All patients	COPD	RD	NMD	OHS/OL
N	231	98	49	15	69
Gender (f/m)	86/145	28/70	28/21 **	5/10	25/44
Age (yrs)	62.9 ± 10.2	64.6 ± 8.2	63.2 ± 12.0	62.8 ± 7.1	60.3 ± 11.4 ***
BMI (kg/m^2^)	33.2 ± 9.9	29.4 ± 7.0	29.3 ± 5.6	28.9 ± 10.0	42.3 ± 9.9 ***
Ventilator use (h/d)	6.76 ± 2.52	6.70 ± 2.81	7.16 ± 2.11	7.20 ± 2.37	6.47 ± 2.39
Duration of HMV (months)	28.9 ± 8.8	29.6 ± 28.3	45.2 ± 36.9 *	27.4 ± 25.5	32.1 ± 25.2
Hb (g/dL)	13.7 ± 1.6	13.9 ± 1.7	13.7 ± 1.5	13.7 ± 1.2	13.5 ± 1.7
Leukocyte number (10^3^/μL)	8.97 ± 3.13	10.1 ± 3.5	7.5 ± 2.3 ***	7.2 ± 2.5*	8.8 ± 2.6 *
Heart disease, N (%)	65 (28.1)	29 (29.6)	11 (22.4)	5 (33.3)	20 (29.0)
Diabetes, N (%)	41 (17.7)	20 (20.4)	4 (8.1)	2 (13.3)	15 (21.7)
Hyperlipidaemia, N (%)	27 (11.7)	11 (11.2)	7 (14.2)	1 (6.7)	8 (11.6)
Hypertension, N (%)	121 (52.4)	42 (42.8)	22 (44.9)	7 (46.7)	50 (72.5) ***
FEV_1 _(L)	1.25 ± 0.71	0.85 ± 0.26	0.89 ± 0.34	1.25 ± 0.58 *	2.08 ± 0.68 ***
FEV_1 _(%pred)	48.7 ± 22.3	32.7 ± 8.7	44.0 ± 14.6 **	50.3 ± 24.0 **	73.9 ± 17.1 ***
FEV/IVC (%)	61.9 ± 17.8	45.8 ± 9.2	74.3 ± 9.9 ***	87.4 ± 17.2 ***	70.3 ± 11.4 ***
IVC (L)	2.05 ± 0.95	1.91 ± 0.55	1.19 ± 0.44 ***	1.44 ± 0.66 **	2.98 ± 0.94 ***
IVC (%pred)	70.0 ± 21.2	56.3 ± 13.9	48.4 ± 16.5 **	46.2 ± 22.6 *	82.8 ± 16.3 ***
pH	7.42 ± 0.04	7.42 ± 0.03	7.42 ± 0.04	7. 43 ± 0.03	7.43 ± 0.04
PaO_2 _(mmHg)	68.0 ± 16.0	66.8 ± 16.2	71.1 ± 18.1	69.2 ± 19.1	67.2 ± 13.3
PaCO_2 _(mmHg)	44.2 ± 6.8	46.4 ± 6.7	44.7 ± 6.6	40.5 ± 6.0**	41.6 ± 6.2 ***
BE (mmol/L)	3.93 ± 3.04	4.76 ± 3.33	3.84 ± 2.57	2.86 ± 2.06*	3.06 ± 2.81 **

Data are shown as mean ± SD. Characteristics of patients with COPD were compared with those of patients with other diseases using the unpaired t-test, Mann-Whitney U-test, or Fisher's exact test. P-values for individual tests were *p < 0.05; **p < 0.017 (corresponding to an overall p < 0.05 using a Bonferroni correction for three comparisons); ***p < 0.001. *Definition of abbreviations: *COPD = chronic obstructive pulmonary disease; RD = restrictive disease; NMD = neuromuscular disease; OHS = obesity-hypoventilation syndrome; OL = overlap syndrome: BMI = body-mass index; HMV = home mechanical ventilation; Hb = haemoglobin; FEV_1 _= forced expiratory volume in one second; IVC = inspiratory vital capacity; PaO_2 _= arterial oxygen tension; PaCO_2 _= arterial carbon dioxide tension; BE = base excess. Blood gas parameters were obtained while patients were breathing room air (n = 147) or their usual oxygen flow (n = 84).

Home ventilators were set at a volume- or pressure-cycled assist-controlled mode. Patients were ventilated via nasal (95.2%) or full-face (3.1%) mask or tracheostomy (1.7%). Median (quartiles) expiratory pressure was 4 (3; 5) cmH_2_O, inspiratory pressure 20 (18; 24) cmH_2_O, and respiratory frequency 19 (16; 22)/min. Patients had spent 25.1 (8.1; 49.7) months on nocturnal HMV prior to enrolment; ventilator use was 6.8 (4.8; 8.3) h/day. Long term oxygen therapy (LTOT) was administered in 81% of patients (96% COPD, 70% RD, 53% NMD, 74% OHS/OL).

SRI-SS differed significantly between groups (ANOVA, p < 0.001; Table 2), specifically between COPD (52.5 ± 15.6) and non-COPD (67.6 ± 16.4; p < 0.001). According to Newman-Keuls there were two homogeneous groups for SRI-SS: COPD showed similar values as NMD, and RD similar values as OHS/OL. SRI-SS was not different between OL and OHS (p = 0.491). Values did also not differ between male and female or non-invasively ventilated and tracheostomised (n = 4) patients, nor depend on the fact whether nasal or face masks were used nor whether patients had LTOT nor not. The subdomains showed very similar results as the SRI-SS (Table 2).

**Table 2:** Results of SRI questionnaire within subgroups.

SRI – subscore	All (n = 231)	COPD (n = 98)	RD (n = 49)	NMD (n = 15)	OHS/OL (n = 69)
Respiratory complaints	61.2 ± 19.8	50.9 ± 17.5	65.8 ± 17.7***	59.4 ± 13.9*	72.3 ± 18.7***
Physical functioning	49.4 ± 24.9	38.2 ± 21.6	55.0 ± 24.7**	33.6 ± 18.0	64.2 ± 22.0***
Attendant symptoms/sleep	63.6 ± 19.0	58.9 ± 18.2	65.8 ± 19.4	59.0 ± 16.5	69.4 ± 19.1***
Social relationship	71.9 ± 18.5	64.9 ± 19.9	75.8 ± 14.6**	68.8 ± 14.3	79.3 ± 16.5***
Anxiety	62.5 ± 23.6	51.8 ± 21.6	66.9 ± 24.2**	61.7 ± 19.1	74.1 ± 20.7***
Psychological wellbeing	62.9 ± 19.6	55.7 ± 19.2	68.3 ± 19.0**	53.6 ± 12.7	70.9 ± 17.4***
Social functioning	58.2 ± 23.3	47.3 ± 20.9	66.2 ± 23.7***	50.3 ± 16.6	68.8 ± 21.5***
Summary score	61.2 ± 17.7	52.2 ± 15.6	66.2 ± 17.2***	55.3 ± 9.2	71.3 ± 15.7***

*Definition of abbreviations: *SRI = Severe Respiratory Insufficiency Questionnaire; COPD = chronic obstructive pulmonary disease; RD = restrictive disease; NMD = neuromuscular disease; OHS = obesity-hypoventilation syndrome; OL = sleep apnoea syndrome. Mean values and SD of HRQL are given, and differences between COPD and the other groups were compared by the t-test, as values were normally distributed within groups. *p < 0.05; **p < 0.017 (overall p < 0.05 according to Bonferroni correction); ***p < 0.001.

### Long-term survival and prognostic factors in the total population

In the total population (n = 231), the mean observation time was 28.9 ± 8.8 months, ranging from 0.2 (death one week after discharge) to 45.8 months. During the study period, 44 patients died (overall mortality 19.1%; COPD 31.6% (n = 31), non-COPD 9.8% (OHS n = 7, RD n = 6)), either from respiratory (n = 29; 65.9%), or non-respiratory (n = 3; 6.8%), or not further specified causes (n = 12; 27.3%).

In the total population survival rates (standard error) at 1, 2 and 3 years were 93.1 (1.7), 84.3 (2.4), and 78.4 (3.0) %, respectively. In COPD, the respective values were 85.7 (3.5), 72.4 (4.5) and 65.3 (5.3) %, and in non-COPD 98.5 (1.1), 93.1 (2.2), and 88.1 (3.2) %. Survival differed between COPD and non-COPD (p < 0.001; HR 0.266; 95%-CI 0.139–0.508), but not between NMD, OHS/OL, or RD. The fact whether patients were ventilated via nasal, full-face mask or tracheostomy was not related to survival, similarly as for LTOT.

**Figure 1:**
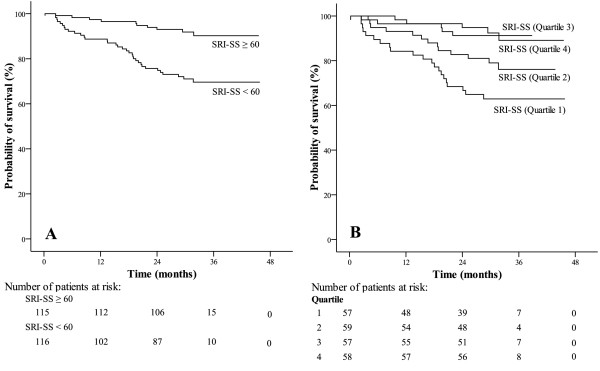
Prognostic value of HRQL in the total population of patients (n = 231) using the median (Panel A; SRI-SS 60.0) as cut-off value (HR 0.262; 95%-CI 0.129–0.530; p < 0.001) or the quartiles (Panel B; 0–49.7, quartile 1; 49.7–60.0, quartile 2; 60.0–74.9, quartile 3; > 74.9, quartile 4; log rank; HR 0.533; 95%-CI 0.394–0.722; p < 0.001).

In univariate analyses, BMI, leukocyte number, base excess (BE), FEV_1 _and FEV_1_/IVC were significantly associated with survival in the total population (Table 3). Neither gender nor comorbidities including heart disease, diabetes, hyperlipidaemia and systemic hypertension, nor medication were related to survival. Regarding SRI, all subscores, with the exception of attendant symptoms and sleep (AS), were predictors of survival (Table 3). Accordingly, SRI-SS was predictive when using the median (Figure 1, panel A) or quartile values (Figure 1, panel B) as cut-off.

**Table 3:** Risk factors according to univariate survival analyses in the total population of patients (n = 231).

Variable	Median (Quartile)	p-value^§^	HR	95%-CI of HR
Disease category ^+^	-	< 0.001	0.266	0.139–0.508
Sex (m/f)	-	0.091	1.803	0.911–3.568
Age (yrs)	65.4 (57.1; 70.4)	0.074	1.745	0.940–3.238
BMI (kg/m^2^)	31.6 (26.9; 38.2**)	0.006	0.420	0.223–0.792
Leukocytes (10^3^/μL)	8.6 (6.6*; 10.6*)	0.002	2.745	1.414–5.332
BE (mmol/L)	3.70 (2.00; 5.50***)	0.027	1.991	1.067–3.715
FEV_1 _(L)	1.03 (0.76; 1.59*)	< 0.001	0.267	0.131–0.544
FEV_1 _(%pred)	42.1 (31.9**; 63.5*)	0.001	0.351	0.179–0.686
IVC (L)	1.84 (1.38; 2.56)	0.112	0.617	0.338–1.125
IVC (%pred)	60.8 (45.9; 76.1*)	0.205	0.665	0.352–1.255
FEV/IVC (%)	62.0 (47.0; 76.0)	0.001	0.950	0.920–0.981
SRI-RC	62.5 (46.9*; 75.0**)	< 0.001	0.280	0.142–0.554
SRI-PF	50.0 (33.3*; 70.0*)	0.002	0.379	0.198–0.724
SRI-AS	64.3 (53.6; 75.0)	0.124	0.629	0.342–1.142
SRI-SR	75.0 (58.3**; 83.3*)	0.002	0.381	0.202–0.718
SRI-AX	65.0 (45.0*; 80.0*)	0.006	0.420	0.223–0.793
SRI-PW	61.1 (50.0**; 77.8)	0.012	0.464	0.251–0.857
SRI-SF	56.3 (40.6*; 75.0*)	0.002	0.374	0.198–0.705
SRI-SS	60.0 (49.7**; 74.9*)	< 0.001	0.262	0.129–0.530

Median values and quartiles are given for the total population, as values were not normally distributed due to the pooling of groups.^+ ^COPD vs. non-COPD. ^§ ^according to univariate survival analyses (log-rank) using the respective median value. Additionally, 25^th ^and 75^th ^percentiles were used. *p < 0.05; **p < 0.01; ***p < 0.001. *Definition of abbreviations: *HR = hazard ratio for survival; CI = Confidence interval; BMI = body-mass index; BE = base excess; FEV_1 _= forced expiratory volume in one second; IVC = inspiratory vital capacity; SRI = Severe Respiratory Insufficiency Questionnaire; RC = Respiratory Complaints; PF = Physical Functioning; AS = Attendant symptoms and Sleep; SR = Social Relationships; AX = Anxiety; WB = Psychological Well-being; SF = Social Functioning. SS = Summary Score.

Stepwise multivariate Cox regression analysis, including the quantitative factors identified in univariate analyses (BMI, leukocytes, BE, FEV_1_, FEV_1_/IVC, SRI-SS) revealed as independent predictors in the total population leukocyte number (HR 2.693, 95%-CI 1.349–5.375; p = 0.005), FEV_1 _(HR 0.313, 95%-CI 0.152–0.644; p = 0.002) and SRI-SS (HR 0.383, 95%-CI 0.186–0.789; p = 0.009). To assess whether the difference in survival between COPD and non-COPD influenced this result, the analysis was repeated by including disease category as binary (COPD versus non-COPD) variable. Again, FEV_1_, SRI-SS and leukocytes were independent risk factors (p < 0.05), whereas disease category did not show any more a significant association in this multivariate analysis (p = 0.192). When disease category was added as variable to each of the other variables in separate analyses, these variables were still predictors in addition to the disease (p < 0.05 each).

### Prognostic factors in COPD patients

When analysing the data of COPD separately (n = 98), FEV_1 _(75^th ^percentile 1.04 L, p < 0.012), BMI (75^th ^percentile 33.9 kg/m^2^, p < 0.009) but neither SRI subdomains nor SRI-SS were associated with survival; also leukocyte number did not reach statistical significance (median 9.6 *10^3^/μL, p = 0.059).

### Prognostic factors in non-COPD patients

In contrast, in non-COPD patients SRI-RC, SRF-PF, SRI-SR, SRI-PW and SRI-SF using the 25^th ^percentile (p < 0.01, each), and SRI-RC, SRI-SR, SRI-PW, SRI-SF using the 50^th ^percentile (p < 0.05 each), as well as SRI-SS (25^th ^and 50^th ^percentile; p = 0.009 and p = 0.039 respectively) were linked to survival. Additionally leukocyte number was a predictor of long-term survival (75^th ^percentile 10.0 *10^3^/μL; p = 0.012). When the analysis was repeated in non-COPD patients by excluding NMD, the results regarding SRI subdomains and SRI-SS became even more pronounced despite the reduction in sample size, while leukocytes remained as a predictor (50^th ^percentile 7.8 *10^3^/μL; p = 0.034; 75^th ^percentile 10.1 *10^3^/μL; p = 0.048).

## Discussion

The present study indicated that in patients with CHRF treated with HMV, specific HRQL assessed by the SRI questionnaire was an independent predictor of long-term survival. Especially in non-COPD patients who showed a favourable survival compared to COPD, the SRI summary score and most of the subscores were associated with prognosis. In COPD, the predictive power of SRI for survival was inferior compared to biological measures. These results suggest that self-reported health-status reflects disease characteristics that are relevant for prognosis and not contained in physiological measures.

HMV is considered to improve long-term survival in various diseases presenting with CHRF [15,21,27,28] but only few useful measures are currently known for monitoring CHRF during treatment with HMV [2,3,15,17]. To our knowledge, the present study is novel in assessing disease-specific HRQL in relation to long-term survival in these patients. The observation period covered 2–4 years in a large population of COPD, NMD, RD, or OHS/OL. Irrespective of their different aetiologies, patients constituted a fairly homogeneous group, as those treated with HMV for < 3 months were excluded and in 98% of patients ventilation was non-invasive. Noteworthy enough, the mode of ventilation had no impact on survival.

Most studies on the impact of HRQL in CHRF have utilized non-specific measures such as the Sickness Impact Profile (SIP) [19,20], Health Index (HI) [19], Sense of Coherence (SOC) [19], Nottingham Health Profile (NHP), or SF-36 [21]. Moreover, these studies predominantly enrolled patients with restrictive disease, such as NMD, post-polio syndrome, or kyphoscoliosis. Recent data, however, indicate that COPD became a major indication for HMV, representing a proportion of 34% [1]. As in our study 42% of patients had COPD, our results seem to reflect very well the frequency distribution regarding the current clinical use of this treatment.

Patients with CHRF suffer from functional impairment and respiratory symptoms, but specifically from the sequels of CHRF such as daytime sleepiness, morning headache and sleep disturbances. To account for their specific conditions of their daily life, the Maugeri Foundation Respiratory Failure item set (MRF-28) has been developed [18] and shown to be useful. This referred primarily to COPD, as the study included only 17 patients with kyphoscoliosis. More recently, the SRI questionnaire has been validated in a large population of patients (n = 226) for the assessment of HRQL in CHRF and HMV [22]. Based on this it has also been employed in the present investigation. In line with previous data [19-22], we found significant differences in HRQL between disease categories. Regarding the SRI summary score, HRQL was most impaired in COPD or NMD, and showed highest values in OHS/OL.

To assess the role of the SRI relative to other measures, we first analyzed the data of the total population of patients. Specific HRQL was an independent predictor of survival, in addition to FEV_1 _and systemic inflammation in terms of leukocyte number. Thus, these results indicate that in patients with CHRF and HMV, HRQL provides valuable information for long-term survival beyond that of biological predictors [2,3]. In a large study of 446 patients with end-stage lung disease of different aetiologies receiving LTOT and/or HMV, C-reactive protein (CRP) and BMI were revealed as important prognostic factors [3]. Instead of CRP we evaluated leukocyte number which was similarly associated with mortality, suggesting a link to systemic inflammation, as in cardiovascular diseases [15,29]. Surprisingly, the prognostic value of BMI was weak in our study, presumably as the BMI-associated risk in OHS or OL is different from that in COPD [15]. In line with this, BMI was predictive for long-term survival when patients with COPD were analyzed separately.

In a second step we evaluated SRI separately in the two major groups of patients comprising a sample size sufficient for survival analysis (COPD and non-COPD). This was even more relevant, as HRQL differs between diseases in CHRF [19,20,22], in accordance with our data. It thus might be suspected that the association with HRQL was due to differences of survival rate between diseases that paralleled those of HRQL. Indeed, and in line with the literature [21,30], mortality was highest in COPD, while it was lower and rather similar in the other diseases. Irrespective of this, the adjusted multivariate analysis suggested that the differences in survival between groups were primarily attributable to the differences in the prognostic measures. Moreover, when patients with COPD were excluded, SRI-SS and nearly all subdomains were highly predictive for survival. In NMD, HRQL was impaired similarly as in COPD; thus these patients were not quite comparable to the other non-COPD groups. Omission of NMD even improved the results regarding the association between long-term survival and HRQL. It seems likely that the low level of HRQL as well as elevated mortality in COPD indicated the impact of multimorbidity that is often present in this disease.

The weak association between HRQL and long-term survival in COPD may have been the result of different factors. The average score of some subdomains in this group was possibly too low to provide sufficient range for the assertion of significant associations. Clinical experience also shows that HMV is often perceived as cumbersome in COPD, impairing HRQL. In fact, the predictive value of HRQL for long-term survival in COPD is still controversial. While the COPD-specific St. George Respiratory Questionnaire (SGRQ) was associated with long-term mortality across different severities of airflow limitation [7,8], the Chronic Respiratory Questionnaire (CRQ) was not related to 3-year survival after pulmonary rehabilitation in a population comprising mainly COPD [31]. Noteworthy enough, the CRQ does not cover physical disability [7], an important prognostic factor in COPD [5]. In line with this, the present investigation showed a tendency towards an association between the SRI subdomain "physical functioning" and mortality in COPD (Kaplan-Meier; p = 0.10, data not shown). Taken together, the findings suggest that the prognostic value of a questionnaire in patients with CHRF much depends on disease-specific features, as reflected in different relative weights of subdomains. It is reassuring in this respect that dyspnoea scores, which comprise a grading of functional capacity or respiratory symptoms, such as the Modified Medical Research Council (MMRC) Score, Borg Scale or Breathing Problems Questionnaire (BPQ), appear particularly informative with regard to disease severity and its relation to mortality [6,31].

In non-COPD patients, low HRQL was related to increased mortality. Accordingly, in COPD mortality was high and HRQL low. In this respect there was as link between HRQL and mortality in all diseases associated with CHRF and HMV although in COPD biological measures dominated. Apparently, self-reported health status provides a comprehensive picture which under these circumstances is more informative beyond biological indices. Indeed, correlations between HRQL and lung function were weak in the majority of cases [32,33].

The present study, though being prospective, was subject to some limitations. The number of patients included was large but still small compared to the number of deaths. It is, however, elucidating that SRI turned out to be predictive particularly in the group of non-COPD patients despite the lower mortality rate that limited the power of the study. As blood gas values were mostly assessed during LTOT, the assessment of their value, especially for arterial oxygen tension, was probably biased. Moreover, 6-min walk distance (6-MWD), an important indicator in COPD, was not included, as it could not be assessed in all patients due to inability or paralysis. However, it might be of interest that there is evidence for an association between 6-MWD and subjective factors [33] and that therefore part of the predictive value of 6-MWD might have been contained in the SRI. Of course, HRQL can be no more than one factor in the multivariate panel determining the clinical state and prognosis of patients with CHRF.

## Conclusion

In summary, our findings provided evidence that in patients with CHRF and current HMV disease-specific HRQL as quantified by the SRI questionnaire was associated with long-term survival but that its predictive value depended on the underlying disease. Thus, disease- specific HRQL bears additional information for long-term outcome beyond that supplied by physiological measures. This information might be useful for the assessment and routine monitoring of patients with CHRF, rendering the picture of impaired health more precise through inclusion of the patients' perception.

## Abbreviations

BE: Base excess;

BMI: Body mass index;

BPQ: Breathing Problems Questionnaire;

COPD: Chronic obstructive pulmonary disease;

CHRF: Chronic hypercapnic respiratory failure;

CRQ: Chronic Respiratory Questionnaire;

FEV_1: _Forced expiratory volume in one second;

HI: Health-Index;

HRQL: Health-related quality of life;

HMV: Home mechanical ventilation;

LTOT: Long-term oxygen therapy;

MMRC: Modified Medical Research Council;

MRF-28: Maugeri Foundation Respiratory questionnaire;

NMD: Neuromuscular disorder;

NHP: Nottingham Health Profile;

OHS: Obesity-hypoventilation syndrome;

OL: Overlap syndrome;

PaCO_2: _Arterial carbon dioxide tension;

PaO_2: _Arterial oxygen tension;

RD: Restrictive disease;

SD: Standard deviation;

SIP: Sickness Impact Profile;

SGRQ: St. George Respiratory Questionnaire;

SOC: Sense of Coherence;

SRI: Severe respiratory insufficiency questionnaire;

SRI-RC: SRI subdomain respiratory complaints;

SRI-PF: SRI subdomain physical functioning;

SRI-AS: SRI subdomain attendant symptoms and sleep;

SRI-SR :SRI subdomain social relationship;

SRI-AX: SRI subdomain anxiety;

SRI-PW: SRI subdomain psychological wellbeing;

SRI-SF: SRI subdomain social functioning;

SRI-SS: SRI summary score;

6-MWD: Six-minute walk distance;

VC: Vital capacity.

## Competing interests

The author(s) declare that they have no competing interests.

## Authors' contributions

SB designed the study, performed part of the data evaluation and participated in writing the manuscript. APH collected a large part of the data, performed part of the data evaluation and helped in writing. RAJ participated in the statistical evaluation of the data, their interpretation and in writing the manuscript. KS and FH collected part of the data and participated in interpreting the data. MP enabled the realization of the study, supervised its performance and participated in data interpretation. All authors had full access to all the data in the study and take responsibility for the integrity of the data and the accuracy of the data analysis.
